# Implementing Cognitive Remediation Therapy (CRT) in the routine treatment of schizophrenia

**DOI:** 10.1192/j.eurpsy.2025.153

**Published:** 2025-08-26

**Authors:** T. Wykes

**Affiliations:** Psychology, Institute of Psychiatry, Psychology and Neuroscience, London, United Kingdom

## Abstract

**Background:**

Despite rigorous evidence of the benefits, costs and savings and mentions in treatment guidance, cognitive remediation access is still sparse. Providers are often confused by disagreements about the strength of the benefits evidence, some think it is a game not a treatment and others do not consider that cognition should be a treatment target. These are all issues that were present in the literature at least ten years ago.

**Aim:**

To identify the steps to widespread implementation of this beneficial intervention

**Method:**

Drawing on the literature and data from a large UK adaptive randomised control trial, we describe three: (i) the barriers and facilitators for the implementation into first episode services, (ii) cost effectiveness and (iii) training rollout and competency.

**Results:**

(i) Clinicians need to understand the relevance of cognition and be aware of effective interventions, (ii) Despite evidence of efficacy a therapist seems important for engagement and leads to cost effective therapy and (iii) online therapy can improve competencies for delivering cognitive therapy and but commitment to completing therapy is related to management commitment.

**Conclusion:**

All the implementation issues can be overcome but we still need to understand the non-specific effects of cognitive remediation as well as the specific if we are to provide a formulation-based approach. Clinicians need to know that cognitive remediation is not **“**brain training**”** but is a holistic therapy that involves an active therapist providing motivation support, and who helps to mitigate the impact of cognitive difficulties through metacognition to develop awareness of cognitive approaches to problems.

**Disclosure of Interest:**

T. Wykes Grant / Research support from: NIHR grant 2.2m

